# Entrapment of the extensor pollicis longus tendon in a pediatric Smith fracture: A case report

**DOI:** 10.1097/MD.0000000000018186

**Published:** 2019-11-27

**Authors:** Yoon Min Lee, Yun Hwan Kim, Yuna Kim, Yoo Joon Sur

**Affiliations:** Department of Orthopedic Surgery, College of Medicine, The Catholic University of Korea, Seoul, Republic of Korea.

**Keywords:** distal radius fracture, extensor pollicis longus, Smith fracture, tendon entrapment

## Abstract

**Rationale:**

Most pediatric distal radius fractures are effectively treated nonoperatively; however, operative intervention is indicated in patients with open and highly unstable fractures, in those with concomitant neurovascular injuries and in patients whom soft tissue interposition between fracture fragments precludes anatomical reduction. Notably, soft tissue interposition between fracture fragments is diagnostically challenging. Surgeons must be mindful of this rare complication for early detection and prompt treatment.

**Patient concerns:**

A 14-year-old boy presented to the emergency department with left wrist pain after falling from a bicycle. Plain radiography and computed tomography revealed a displaced Smith fracture, which was irreducible by closed reduction, necessitating open reduction and volar plate fixation. The patient reported inability to extend his thumb at his 6-week postoperative follow-up visit.

**Diagnosis:**

Ultrasonography showed extensor pollicis longus (EPL) tendon entrapment near the fracture site.

**Interventions:**

A second operation was performed 10 weeks after the first surgery, and intraoperative exploration revealed EPL tendon entrapment. The EPL tendon was torn to shreds; therefore, extensor indicis proprius tendon transfer was performed for EPL tendon reconstruction.

**Outcomes:**

The patient's thumb motion was completely restored after the second operation.

**Lessens:**

EPL tendon entrapment in a pediatric Smith fracture is rare. Signs of EPL tendon entrapment include inability to perform active thumb extension, dorsal wrist pain radiating along the course of the EPL tendon, which is exacerbated by thumb flexion, a tenodesis effect elicited on thumb examination, and difficulty in anatomical fracture reduction. Surgical exploration of the EPL tendon is warranted in patients presenting with any of these signs following attempted reduction of a Smith fracture.

## 1 Introduction

Distal radius fractures are among the most common fractures in children and account for approximately 20% of all pediatric fractures. Nearly all distal radius fractures are displaced dorsally. Although most are effectively treated nonoperatively, operative treatment is indicated in patients with open and highly unstable fractures, in those with concomitant neurovascular injuries, and in patients in whom soft tissue interposition between fracture fragments precludes an anatomical reduction. Patients with a Colles fracture most commonly present with entrapment of the volar periosteum and pronator quadratus, whereas those with a Smith fracture show entrapment of the extensor retinaculum and extensor tendons.^[1]^ Although not anatomically perfect, acceptable reduction can be obtained despite soft tissue interposition. Notably, soft tissue interposition between fracture fragments is diagnostically challenging. Limitation of finger motion caused by tendon interposition between fracture fragments is often indistinguishable from painful limitation of movements caused by the fracture itself. Furthermore, finger motion maybe preserved despite tendon interposition.^[2]^ We report the case of 14-year-old boy who sustained a Smith fracture with EPL tendon entrapment in whom diagnosis of tendon entrapment was delayed. Written informed consent was obtained from the patient for the publication of this case report and accompanying images. Ethical approval for this study was waived by the Ethics Committee of our hospital considering the nature of this study (case report).

## 2 Case report

**Figure 1:**
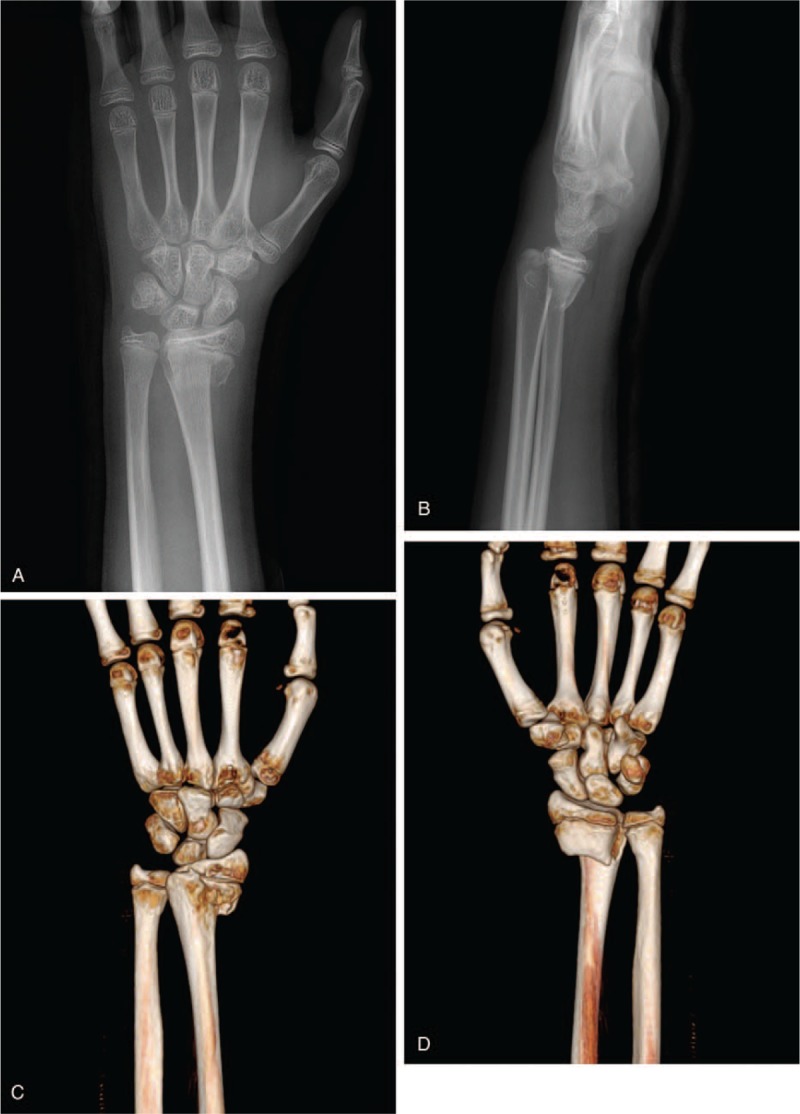
Initial plain radiographs (A, B) and 3D-CT reconstruction images (C, D) showing a displaced Smith fracture with a Salter-Harris type 2 growth plate injury.

**Figure 2:**
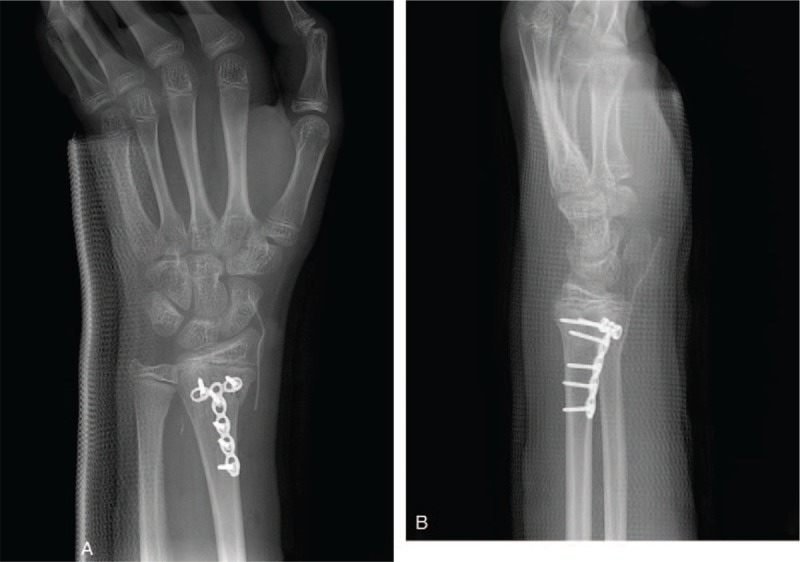
Postoperative posteroanterior and lateral plain radiographs showing volar plate fixation of the Smith fracture.

**Figure 3:**
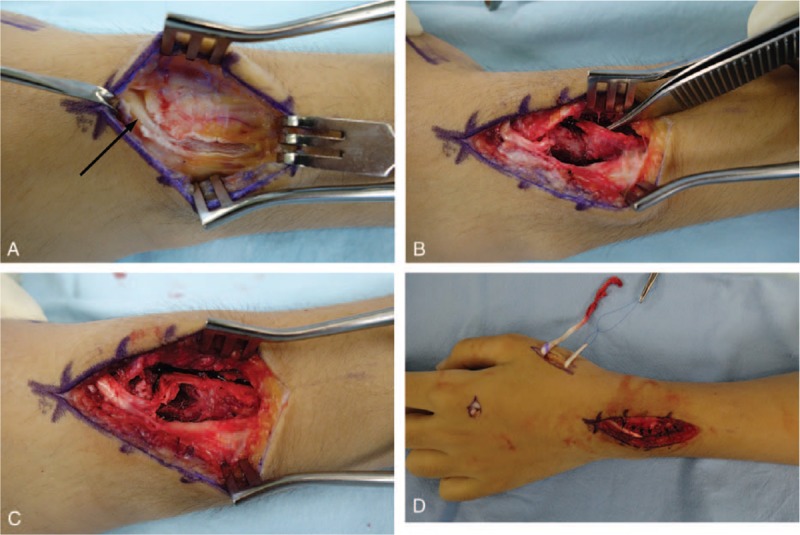
Intraoperative photographs showing the EPL tendon entrapment. (A) The distal portion of the EPL tendon is visualized within the third extensor compartment (black arrow). However, when traced proximally, the tendon disappears just proximal to the Lister tubercle. (B) The extensor retinaculum and dorsal periosteum were divided and the proximal portion of the EPL tendon was identified. The tweezers indicate the proximal portion of the EPL tendon. (C) Image showing the dorsoradial cortex of the distal radius has been removed, and the entrapped EPL tendon is exposed. (D) The EPL tendon is reconstructed with the EIP transfer.

**Figure 4:**
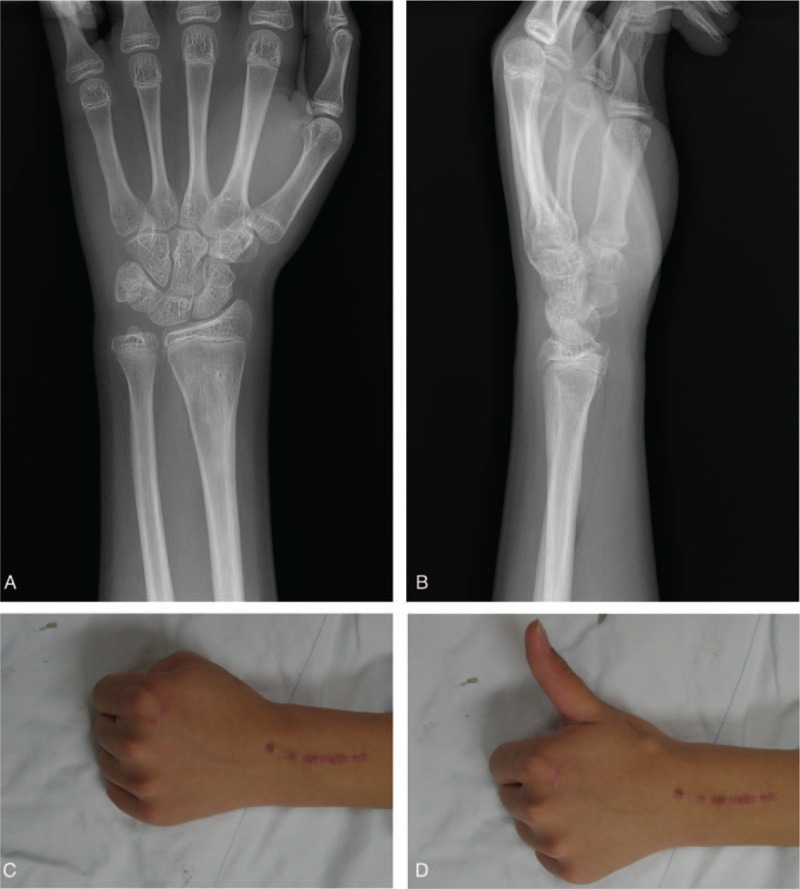
Plain radiographs (A, B) and photographs (C, D) obtained 10 months after the initial injury showing complete union of the fracture and full flexion and extension of the thumb.

A 14-year-old boy presented to the emergency department with left wrist pain after falling from a bicycle. Physical examination revealed edema, bruising, and a reverse bayonet deformity of his left wrist. Plain radiography and computed tomography revealed a displaced Smith fracture (Fig. 1). The distal fracture fragment showed volar and radial displacement. Closed reduction was attempted under conscious sedation for a suspected simple displaced fracture. However, the fracture was irreducible, and the patient was admitted for surgical treatment using Kirschner wire fixation. We planned to treat the fracture by Kirschner wire fixation. Closed reduction was re-attempted under general endotracheal anesthesia; however, this attempt failed, necessitating open reduction and internal fixation. The fracture site was exposed using the standard flexor carpi radialis approach, and the volarly displaced fracture was reduced. Unfortunately, the distal fracture fragment tended to slip volarly, and Kirschner wire fixation was impossible. However, despite a significant degree of difficulty, successful anatomical reduction and internal fixation were achieved with volar buttress plating (Fig. 2). Postoperatively, although the patient complained of moderate pain with thumb motion, active motion of his thumb and other fingers was restored. Active and passive range-of-motion exercises of all fingers were introduced the day following the operation. After 4-week immobilization in a short-arm cast, adequate fracture union was confirmed by plain radiography, and wrist motion was permitted. At his 6-week postoperative follow-up, the patient reported inability to extend his left thumb. Ultrasonography revealed EPL tendon entrapment near the fracture site. A second operation was performed 10 weeks after the first and intraoperative exploration revealed EPL tendon entrapment (Fig. 3). The proximal portion of the EPL tendon was incarcerated at the dorsoradial aspect of the fracture site, and the distal portion of the EPL tendon was within the third extensor compartment just proximal to the Lister tubercle. The dorsoradial cortex of the distal radius was removed to expose the incarcerated portion of the EPL tendon. The EPL tendon was torn to shreds; therefore, extensor indicis proprius (EIP) tendon transfer was performed for EPL tendon reconstruction. The patient's thumb motion was completely restored after 5-week immobilization. Hardware was removed 6 months after the first operation. Plain radiographs obtained 10 months after the initial injury showed no evidence of pathological changes, and the patient was able to engage in daily activities without any symptoms (Fig. 4).

## 3 Discussion

EPL tendon rupture is a well-described complication of a Colles fracture with incidence rates of 0.2% to 5.0%.^[3]^ It occurs more commonly in cases of nondisplaced or minimally displaced fractures, particularly when the extensor retinaculum is intact.^[3]^ Mechanical attrition and ischemic insult have been suggested as possible etiopathogenic contributors. In contrast, EPL tendon entrapment or rupture associated with a Smith fracture is relatively rare. In patients with Smith fractures, the EPL tendon may be injured directly by sharp edges of the proximal fracture fragment or it could be incarcerated between fracture fragments. The mechanism that contributes to EPL tendon injury in cases of a Smith fracture differs from that observed in cases of a Colles fracture. EPL tendon entrapment in a Smith fracture was first reported by Hunt in 1969.^[4]^ Owing to its rarity, only a few cases have described this condition since the publication of Hunt's report. A review of cases published in the English literature revealed 6 pediatric cases of EPL tendon entrapment associated with a Smith fracture.^[5–10]^ The following anatomical features predispose the EPL tendon to entrapment in cases of a Smith fracture:

(1) it crosses the wrist joint from a dorsal to volar position in sagittal plane and,
(2) it crosses the wrist joint obliquely in a horizontal plane.

These characteristics make it highly vulnerable to entrapment between the fracture fragments in patients with a Smith fracture presenting with volar displacement of fracture fragments. The mechanism of the EPL tendon entrapment in patients with a Smith fracture was first proposed by Hunt in 1969.^[4]^ Based on a cadaveric model, Hunt proposed that strong axial compression and pronation forces acting on the distal fracture fragment and subsequent supination of the proximal fracture fragment lead to protrusion of the proximal fracture fragment between the extensor carpi radialis brevis (ECRB) tendon and the EPL tendon with dislocation of the EPL tendon between the radius and ulna. Sustained axial compression and rotation forces cause volar displacement of the EPL tendon and eventually its entrapment between the fracture fragments. Karlsson and Appleqvist^[5]^ and Thomas and Kershaw,^[6]^ respectively reported a case each supporting Hunt's theory. However, the mechanism reported by 4 of the 6 published pediatric case report is inconsistent with Hunt's theory.^[7–10]^ The EPL tendon was entrapped by a displaced dorsal metaphyseal cortical bone fragment and at the floor of the third extensor compartment in 2 cases each.^[7–10]^ Furthermore, in the current case, the EPL tendon showed radial displacement with entrapment at the dorsoradial aspect of the fracture site (floor of the second extensor compartment). Upon reviewing the 6 previously published reports and based on our observations in the current case, we conclude that the following three mechanisms contribute to EPL tendon entrapment associated with pediatric Smith fractures. First, radial and volar displacement of the distal fracture fragment causes protrusion of the proximal fracture fragment between the EPL and the extensor digitorum communis, and the EPL tendon is incarcerated between the fracture fragments with a radial orientation relative to its original location. Second, following volar displacement of the distal fracture fragment, the EPL tendon is entrapped at the floor of the third extensor compartment. Third, Ulnar and volar displacement of the distal fracture fragment is accompanied by protrusion of the proximal fracture fragment between the ECRB and the EPL, and the EPL tendon gets trapped between the fracture fragments with an ulnar orientation relative to its original location, similar to that described by Hunt's cadaveric model.

In our case, the EPL tendon was nearly torn by the time its entrapment was diagnosed 6 weeks postoperatively. Although thumb motion recovered following EIP tendon transfer, in our view, the EPL tendon could have been saved if entrapment were to be suspected earlier. As mentioned previously, accurate diagnosis of tendon interposition between fracture fragments is challenging, and among the 6 pediatric cases reported in the literature, immediate diagnosis was possible in only one case.^[5]^ Surgeon must be mindful of this possibility for early detection of this rare complication. Signs of EPL tendon entrapment include inability to perform active thumb extension, dorsal wrist pain radiating along the course of the EPL tendon, which is exacerbated by thumb flexion, a tenodesis effect elicited on thumb examination, and difficulty in achieving anatomical reduction of fracture fragments. Surgical exploration of the EPL tendon is warranted in patients who present with any of these signs after attempted reduction of a Smith fracture.

## Author contributions

**Conceptualization:** Yoo Joon Sur.

**Supervision:** Yoo Joon Sur.

**Writing – original draft:** Yuna Kim, Yun Hwan Kim.

**Writing – review & editing:** Yoon Min Lee.

Yoo Joon Sur orcid: 0000-0003-0090-9970.
